# Supplementary material for: Coming out under fire: The role of minority stress and emotion regulation in sexual orientation disclosure
Source: PLoS One. 2022 May 2;17(5):e0267810. doi: 10.1371/journal.pone.0267810 (PMC9060356; doi:10.1371/journal.pone.0267810)
Supplement: S1 Appendix — (DOCX) [file pone.0267810.s001.docx]

**S1 Appendix. Contents of questionnaire battery.**

- Centers for Epidemiological Studies – Depression Scale (CES-D) [1]
- Social Interaction Anxiety Scale (SIAS) [2]
- GAD-7 [3]
- Short Michigan Alcohol Screening Test (SMAST) [4]
- Drug Abuse Screening Test (DAST-10) [5]
- PTSD Checklist – Civilian Version (PCL-C) [6]
- Sexual Experiences – Short Form Victimization (SES-SFV) [7]
- Emotion Regulation Questionnaire (ERQ) [8]
- Difficulties with Emotion Regulation Scale (DERS) [9]
- Penn State Worry Questionnaire (PSWQ) [10]
- Responses to Positive Affect (RPA) [11]
- Ruminative Responses Scale - Brooding (RRS-B) [12]
- Brief Resilience Scale (BRS) [13]
- Lesbian, Gay, and Bisexual Identity Scale (LGBIS) [14]
- Outness Inventory (OI) [15]
- Everyday Discrimination Scale (EDS) [16]
- Daily Heterosexist Experiences Questionnaire (DHEQ) [17]
- Social Support Behaviors Scale – Socializing, Emotional Support, & Advice/Guidance Subscales (SS-B) [18]

References

1. Radloff LS. The CES-D scale a self-report depression scale for research in the general population. Appl Psychol Meas. 1977;1(3):385–401.

2. Mattick RP, Clarke JC. Development and validation of measures of social phobia scrutiny fear and social interaction anxiety1. Behav Res Ther. 1998;36(4):455–70.

3. Spitzer RL, Kroenke K, Williams JB, Löwe B. A brief measure for assessing generalized anxiety disorder: the GAD-7. Arch Intern Med. 2006;166(10):1092–7.

4. Selzer ML, Vinokur A, Rooijen L van. A self-administered short Michigan alcoholism screening test (SMAST). J Stud Alcohol Drugs. 1975;36(01):117.

5. Skinner HA. The drug abuse screening test. Addict Behav. 1982;7(4):363–71.

6. Weathers FW, Huska JA, Keane TM. PCL-C for DSM-IV. Boston: National Center for PTSD—Behavioral Science Division; 1991.

7. Koss MP, Abbey A, Campbell R, Cook S, Norris J, Testa M, et al. Revising the SES: A collaborative process to improve assessment of sexual aggression and victimization. Psychol Women Q. 2007;31(4):357–70.

8. Gross JJ, John OP. Individual differences in two emotion regulation processes: Implications for affect, relationships, and well-being. J Pers Soc Psychol. 2003;85(2):348–62.

9. Gratz KL, Roemer L. Multidimensional assessment of emotion regulation and dysregulation: Development, factor structure, and initial validation of the difficulties in emotion regulation scale. J Psychopathol Behav Assess. 2004;26(1):41–54.

10. Meyer TJ, Miller ML, Metzger RL, Borkovec TD. Development and validation of the penn state worry questionnaire. Behav Res Ther. 1990;28(6):487–95.

11. Feldman GC, Joormann J, Johnson SL. Responses to positive affect: A self-report measure of rumination and dampening. Cogn Ther Res. 2008;32(4):507–25.

12. Treynor W, Gonzalez R, Nolen-Hoeksema S. Rumination reconsidered: A psychometric analysis. Cogn Ther Res. 2003;27(3):247–59.

13. Smith BW, Dalen J, Wiggins K, Tooley E, Christopher P, Bernard J. The brief resilience scale: assessing the ability to bounce back. Int J Behav Med. 2008;15(3):194–200.

14. Mohr JJ, Kendra MS. Revision and extension of a multidimensional measure of sexual minority identity: The Lesbian, Gay, and Bisexual Identity Scale. J Couns Psychol. 2011;58(2):234–45.

15. Mohr J, Fassinger R. Measuring dimensions of lesbian and gay male experience. Meas Eval Couns Dev. 2000;33(2):66–90.

16. Williams DR, Yu Y, Jackson JS, Anderson NB. Racial differences in physical and mental health socio-economic status, stress and discrimination. J Health Psychol. 1997;2(3):335–51.

17. Balsam KF, Beadnell B, Molina Y. The Daily Heterosexist Experiences Questionnaire: Measuring Minority Stress Among Lesbian, Gay, Bisexual, and Transgender Adults. Meas Eval Couns Dev Sage Publ Inc. 2013 Jan;46(1):3–25.

18. Vaux A, Riedel S, Stewart D. Modes of social support: The social support behaviors (SS-B) scale. Am J Community Psychol. 1987;15(2):209–32.
