# Supplementary material for: Coming out under fire: The role of minority stress and emotion regulation in sexual orientation disclosure
Source: PLoS One. 2022 May 2;17(5):e0267810. doi: 10.1371/journal.pone.0267810 (PMC9060356; doi:10.1371/journal.pone.0267810)
Supplement: S1 Table — (DOCX) [file pone.0267810.s002.docx]

**S1 Table. Descriptive characteristics of disclosure variables by film, emotion regulation condition, and sexual orientation**.

|  | **Lesbian/Gay** (*n* = 75) | | | | **Bisexual** (*n* = 73) | | | | **Other** (*n* = 20) | | | |
| --- | --- | --- | --- | --- | --- | --- | --- | --- | --- | --- | --- | --- |
|  | **Minority Stress** | | **Affirming** | | **Minority Stress** | | **Affirming** | | **Minority Stress** | | **Affirming** | |
|  | **Dist.** | **Imm.** | **Dist.** | **Imm.** | **Dist.** | **Imm.** | **Dist.** | **Imm.** | **Dist.** | **Imm.** | **Dist.** | **Imm.** |
| *n* | 17 | 20 | 23 | 15 | 23 | 15 | 15 | 20 | 4 | 4 | 4 | 8 |
| Disclosure (%) | 76.5 | 65.0 | 73.9 | 93.3 | 34.8 | 60.0 | 60.0 | 85.0 | 25.0 | 50.0 | 100.0 | 62.5 |
| Average total word count | 140.12 (69.76) | 151.20 (63.53) | 131.96 (55.91) | 168.07 (87.60) | 144.96 (55.69) | 148.13 (62.72) | 126.20 (70.47) | 137.90 (59.88) | 138.50 (30.83) | 127.75 (31.89) | 206.00 (55.59) | 108.38 (38.33) |
| Average word count until disclosure | 55.15 (46.61) | 53.15 (42.43) | 48.71 (46.33) | 49.29 (45.04) | 98.00 (61.88) | 82.44 (35.21) | 68.33 (55.35) | 46.00 (39.11) | 96.00 (N/A) | 79.50 (43.13) | 74.25 (70.28) | 78.20 (36.61) |

Dist. = Distancing condition. Imm. = Immersion condition. Mean (Standard deviation). Average word count until disclosure includes only participants who disclosed their sexual orientation during the reflection task.
